# Neonatal overfeeding attenuates acute central pro-inflammatory effects of short-term high fat diet

**DOI:** 10.3389/fnins.2014.00446

**Published:** 2015-01-13

**Authors:** Guohui Cai, Tara Dinan, Joanne M. Barwood, Simone N. De Luca, Alita Soch, Ilvana Ziko, Stanley M. H. Chan, Xiao-Yi Zeng, Songpei Li, Juan Molero, Sarah J. Spencer

**Affiliations:** School of Health Sciences and Health Innovations Research Institute, RMIT UniversityMelbourne, VIC, Australia

**Keywords:** inflammation, microglia, neonatal, obesity, paraventricular nucleus of the hypothalamus (PVN)

## Abstract

Neonatal obesity predisposes individuals to obesity throughout life. In rats, neonatal overfeeding also leads to early accelerated weight gain that persists into adulthood. The phenotype is associated with dysfunction in a number of systems including paraventricular nucleus of the hypothalamus (PVN) responses to psychological and immune stressors. However, in many cases weight gain in neonatally overfed rats stabilizes in early adulthood so the animal does not become more obese as it ages. Here we examined if neonatal overfeeding by suckling rats in small litters predisposes them to exacerbated metabolic and central inflammatory disturbances if they are also given a high fat diet in later life. In adulthood we gave the rats normal chow, 3 days, or 3 weeks high fat diet (45% kcal from fat) and measured peripheral indices of metabolic disturbance. We also investigated hypothalamic microglial changes, as an index of central inflammation, as well as PVN responses to lipopolysaccharide (LPS). Surprisingly, neonatal overfeeding did not predispose rats to the metabolic effects of a high fat diet. Weight changes and glucose metabolism were unaffected by the early life experience. However, short term (3 day) high fat diet was associated with more microglia in the hypothalamus and a markedly exacerbated PVN response to LPS in control rats; effects not seen in the neonatally overfed. Our findings indicate neonatally overfed animals are not more susceptible to the adverse metabolic effects of a short-term high fat diet but may be less able to respond to the central effects.

## Introduction

The developmental origins of health and disease hypothesis suggests the early life period is one of significant vulnerability to programming of physiology by environmental influences (Forsdahl, 1977; Barker and Osmond, 1986; Wadhwa et al., 2009; Spencer, 2012). In particular, early life nutrition is important in programming the development of central and peripheral mechanisms regulating feeding and metabolism, and subsequent susceptibility to overweight or obesity (Spencer, 2012, 2013a,b). As such, perinatal overfeeding has major short- and long-term physiological consequences [e.g., reviewed in (Spencer, 2012, 2013a; Habbout et al., 2013)].

We, and others, have reported neonatal overfeeding in a rodent model leads to accelerated weight gain in early life that persists long-term and is linked with immune and hypothalamic-pituitary-adrenal (HPA) axis dysfunction (Plagemann et al., 1992; Boullu-Ciocca et al., 2005; Spencer and Tilbrook, 2009; Clarke et al., 2012; Smith and Spencer, 2012; Stefanidis and Spencer, 2012). These findings parallel those of human studies where childhood obesity significantly increases the risk an individual will become an obese adult (Whitaker et al., 1997; Stettler et al., 2005; Biro and Wien, 2010). Obese children are also more likely to suffer from immune and HPA axis disturbances as they grow up (Reeves et al., 2008; Lee, 2009; Brune and Hochberg, 2013).

Although there are clear effects of early life nutrition on later susceptibility to overweight/obesity and its pathophysiological sequelae, it is also clear not all overweight children become obese adults (Potter and Ulijaszek, 2013). Similarly, several studies of neonatal overfeeding in rodents have shown that long-term exacerbated weight gain is mild and the animals do not always exhibit hyperphagia or indices of diabetes. For instance, while some studies have demonstrated being suckled in small litters leads to increased food intake in adulthood (Oscai and McGarr, 1978; Rodrigues et al., 2007, 2009), this tends to normalize when corrected for overall body weight (Mozes et al., 2004; Xiao et al., 2007; Stefanidis and Spencer, 2012). Studies also differ in their reporting of whether neonatal overfeeding influences glucose utilization (Plagemann et al., 1999; Xiao et al., 2007). Although some neonatally overfed cohorts show insensitivity to a glucose load in a glucose tolerance test (GTT), differences in glucose uptake into adipocytes, and differences in insulin signaling (Plagemann et al., 1999; Boullu-Ciocca et al., 2005; Rodrigues et al., 2007), indicating a pre-diabetic phenotype, we have seen only mild changes in metabolic parameters (Stefanidis and Spencer, 2012). In this regard neonatal overfeeding appears to result in a moderate predisposition to excessive weight gain, with some indications of diabetic symptoms and significant, but mild, metabolic impairment.

From a pathophysiological perspective, a single adverse event or period is unlikely to be the only factor influencing long-term physiology, however. A sustained high fat diet consumed in adult rodents and humans can lead to excessive weight gain, adiposity, and indices of diabetes such as glucose intolerance and insulin resistance (Rosini et al., 2012). In this study we therefore hypothesized that the mild metabolic phenotype induced by neonatal overfeeding would predispose an animal to more substantial metabolic disturbances later in life if it is also exposed to the “second hit” challenge of a short or medium term high fat diet.

Neonatal overfeeding by suckling rat pups in small litters induces notable but moderate changes in weight gain, feeding, and metabolism throughout life that may be exacerbated by later exposure to high fat diet. However, neonatal overfeeding also causes significant and substantial peripheral and central inflammation, including a pro-inflammatory profile in systemic tissue and the hypothalamus, as well as exacerbated pro-inflammatory response to a neuroimmune challenge with bacterial mimetic lipopolysaccharide (LPS) (Tapia-Gonzalez et al., 2011; Clarke et al., 2012; Ye et al., 2012; Ziko et al., 2014). For this reason we also hypothesized the systemic and central inflammatory profile would be further exacerbated by high fat diet in adulthood in the neonatally overfed rats.

In this study we manipulated litter sizes so that Wistar rats were suckled in litters of four (small litter; SL) or 12 (control litter; CL). The former have greater access to their dam's milk, consume milk that is higher in fat, and show accelerated growth and weight gain that is maintained into adulthood (Fiorotto et al., 1991; Mozes et al., 2004). The pups were weaned onto *ad libitum* normal rat chow, but in adulthood were given either 3 days (3D) or 3 weeks (3W) high fat diet (45% kcal as fat). At the end of this period we assessed changes in weight and indices of diabetes, as well as central and peripheral markers of inflammation. We also examined liver cytokine expression and central neuronal activation in response to i.p. LPS.

## Materials and methods

### Animals

We obtained timed-pregnant Wistar rats from the Animal Resources Centre, WA, Australia. After arrival at the RMIT University Animal Facility, we housed the dams at 22°C on a 12 h light/dark cycle (7 a.m. to 7 p.m.) with free access to pelleted rat chow and water. We conducted all experiments in accordance with the National Health and Medical Research Council Australia Code of Practice for the Care of Experimental Animals. All procedures were approved by the RMIT University Animal Ethics Committee.

### Litter size manipulation

On postnatal day (P) 0, the day of birth, we removed all pups from their dams and randomly fostered them to new dams in litters of 12 (CL; controls) or 4 (SL; neonatally overfed) as we have previously described (Spencer and Tilbrook, 2009; Clarke et al., 2012; Smith and Spencer, 2012; Stefanidis and Spencer, 2012; Ziko et al., 2014). Birth litters included in this study had a range of 8–17 pups, a mean of 13.9 ± 0.36, and mode of 14. No dam received any of her own pups and each new litter was made up of 50% males and 50% females. Excess pups were culled. We have previously shown this litter size manipulation results in SL pups having accelerated growth and weight gain so that they are significantly heavier by around P7 and maintain greater weights into adulthood (Spencer and Tilbrook, 2009; Clarke et al., 2012; Smith and Spencer, 2012; Stefanidis and Spencer, 2012; Ziko et al., 2014).

### Effects of neonatal overfeeding on susceptibility to high fat diet

To test long-term susceptibility to the effects of high fat diet after neonatal overfeeding, we weaned the rats into same-sex littermate pairs on normal rat chow and kept them until P56. At this time they were allocated to the 3D or 3W high fat diet or chow groups. 3D high fat diet (23.5% fat; 45% kcal from fat; Specialty Feeds, WA, Au) was commenced at P74 and 3W high fat diet (as above) was commenced at P56. On P76, i.e., 2 days or 20 days after the onset of the high fat diet, or equivalent in chow fed (4.8% fat) controls, we gave the rats an i.p. glucose tolerance test (GTT). Rats were fasted for 3–4 h prior to testing to standardize basal glucose levels. We then quickly took each rat from its cage and nicked the end off the tail with a sharp razor blade to extract ~20 μL of baseline blood sample into a heparinized capillary tube for measurement of plasma triglycerides. These and liver triglycerides were later determined using calorimetric enzymatic GPO-PAP assays (Roche Diagnostics, IN, USA). Blood samples were kept on ice until the end of the experiment, when they were centrifuged and the plasma aliquots stored at −20°C until assayed. We also measured basal glucose levels at this time using an Accu-Chek Performa blood glucose meter (Roche Diagnostics; Castle Hill, NSW, Au). We then injected each rat with 1.5 g/kg glucose and measured glucose levels at 15, 30, 45, 60, and 90 min after injection.

Two days later, i.e., after 4 or 22 days high fat diet (or chow), the pairs of rats were then randomly allocated into the saline or LPS group. We gave each rat an i.p. injection of LPS (*E. coli*, serotype 026:B6; L-3755; Sigma, St Louis, MO, USA; 100 μg/kg), or pyrogen-free saline. At 120 min after injection, we deeply anesthetized the rats with Lethabarb (~150 mg/kg pentobarbitone sodium, i.p.). We hemisected each rat below the diaphragm and used it for fresh tissue collection and for cardiac perfusion to obtain fixed brains. Thus, we removed livers and male epididymal or female perirenal fat pads. Tissues were weighed and snap-frozen in liquid nitrogen. For the brains, we perfused the rats transcardially with phosphate buffered saline (PBS; 4°C, pH 7.4) followed by 4% paraformaldehyde in PBS (4°C, pH 7.4). We then removed the brains and post-fixed them for 4 h in the same fixative before placing them in cryoprotectant with 20% sucrose in PBS (4°C). We cut forebrains into 30 μm coronal sections using a cryostat. All experiments were initiated between 0900 and 1200 h to limit potential effects of circadian rhythms on any parameters measured.

### Inflammatory gene expression

To assess changes in peripheral markers of inflammation, we measured mRNA expression levels of the free fatty acid and LPS receptor, toll-like receptor 4 (TLR4), downstream transcription factor, nuclear factor κ B (NFκ B), as well as representative pro- and anti-inflammatory cytokines, interleukin (IL)-10, tumor necrosis factor (TNF)α, IL-1β, and IL-6 in the liver and adipose tissues. We isolated RNA from our snap-frozen liver and fat samples using QIAzol and an RNeasy purification kit (QIAGEN, Valencia, CA, USA). The extracted RNA (1 μg) was transcribed to complementary DNA with an iScript cDNA synthesis kit; (Bio-Rad Laboratories, Hercules, CA, USA), following the manufacturer's instructions. We then performed rt-PCR with Taqman Gene Expression Assays (Applied Biosystems, Mulgrave, Vic, Au). We measured fold differences in target mRNA expression with the δ-cycle threshold method by comparison with the housekeeping gene, 18S (Livak and Schmittgen, 2001; Schmittgen and Livak, 2008). Data are expressed as mRNA relative fold change as described previously (Mouihate et al., 2010; Clarke et al., 2012; Spencer et al., 2012).

### Liver cytokine expression

To further assess changes in peripheral markers of inflammation, we examined concentrations of a number of pro- and anti-inflammatory cytokines in the liver using a Bio-Plex assay allowing multiple analytes to be assessed in one sample. Liver samples were lysed using Bio-Plex cell lysis kit (Bio-Rad) according to the manufacturer's instructions. The total protein concentration of the lysates was determined using the bicinchoninic acid (BCA) assay (Pierce™ BCA Protein Assay Kit, Thermo Scientific). Samples were then diluted in Bio-Plex Sample Diluent (containing 0.5% BSA) and assayed in a final concentration of 500 ug/mL using a magnetic beads-based Bio-Plex Pro rat TH1/TH2 12-Plex (Bio-Rad) assay. The assays were performed using the Bio-Plex MAGPIX™ instrument and the data were analyzed using Bio-Plex Manager Software 6.1 (Bio-Rad). Female IL-13, granulocyte macrophage colony-simulating factor, and interferon gamma were not detectable and these were low and not significantly different between groups in the males, so are not reported here.

### Brain microglia and neuronal responses to immune challenge

To assess the influence of early life overfeeding and adult high fat diet on central inflammation, we immunolabelled sections through the hypothalamus for ionized calcium-binding adapter molecule-1 (Iba-1; a marker for microglia/macrophages), seen as amber staining or Fos (a marker of neuronal activation), seen as a black nuclear deposit, as previously described (Spencer et al., 2004a,b; Mouihate et al., 2010; Ziko et al., 2014). Briefly, we incubated separate one-in-five series of forebrain sections from each animal in primary antibody overnight at 4°C (Iba-1, 1:1000; rabbit; Wako Chemicals USA Inc., Richmond, VA, USA or Fos, 1:10,000; rabbit; Santa Cruz Biotechnology, Santa Cruz, CA, USA). This was followed by secondary antibody (1.5 h; 1:200, Iba-1, 1:500, Fos; biotinylated anti-rabbit; Vector Laboratories, Burlingame, CA, USA) and an avidin-biotin horseradish peroxidase (HRP) complex (ABC; 45 min; Vector Elite kit; Vector). The sections were then incubated in diaminobenzidine (DAB) with (black; Fos) or without (amber; Iba1) nickel and colbalt, to visualize the HRP activity. The reactions were terminated once an optimal contrast between specific cellular and non-specific background labeling was reached. Randomly selected brains from each of the treatment groups were processed at the same time in batches. Sections were then air-dried, dehydrated in a series of alcohols, cleared in histolene, and coverslipped.

### Cell counts

An experimenter blinded to treatment condition assessed the sections for differences in numbers of cells with Iba-1 labeling and in density of Iba-1 labeling using photomicrograph images imported into image analysis software Image J (National Institutes of Health, Bethesda, MD, USA), as we have previously described (Beynon and Walker, 2012; Radler et al., 2014; Ziko et al., 2014). Briefly, we took all photomicrograph images from an Olympus upright microscope (Olympus BX41; Olympus, Melbourne, Vic, Au) with a 20 times objective lens using an Olympus DP72 digital camera (Olympus) and LabSens image capture software v1.6 (Olympus) software. Images were taken at 4140 × 3096 pixel density. They were then imported into and processed using Image J. We auto-subtracted background and converted each image to 16 bit for analysis, then cropped each image to take a representative 1602 × 1602 pixel sample from each region of interest within each section. We then assessed numbers of Iba-1-positive cells and density of staining using the thresholding method, as described (Beynon and Walker, 2012; Radler et al., 2014; Ziko et al., 2014), in the paraventricular nucleus of the hypothalamus (PVN; ~1.80 and 1.95 mm caudal to bregma) and in the arcuate nucleus (ARC; ~2.04 to −3.09 mm relative to bregma). Brain regions were identified according to the Paxinos and Watson Rat Brain Atlas (Paxinos and Watson, 2009). For each region, we sampled the left and right sides across two sections of the PVN and five sections of the ARC, 150 μm apart. We saw no differences between left and right hemispheres or rostrocaudal level for any of the regions, so we then took the sum of the images as our sampled result.

An experimenter, blinded to the group treatments, also carried out counts of cells positive for Fos-immunoreactivity in the PVN over two sections (~1.80 and 1.95 mm caudal to bregma), in the dorsal (d) and ventral (v) bed nucleus of the stria terminalis (BNST) over four sections (~0.24 to −0.36 mm relative to bregma), in the medial preoptic area (MPOA) and vascular organ of the laminar terminalis (OVLT) over two sections (~0.36 and 0.51 mm rostral to bregma) and in the ventromedial (VM) POA over two sections (at and 0.15 mm caudal to bregma).

### Data analysis

We compared pre-weaning body weights between CL and SL rats using an analysis of variance (ANOVA) with repeated measures, with litter size as the between factor and age as the repeated measure. When a significant interaction was found between litter size and age we performed Student's unpaired *t*-tests for each time point. We compared adult parameters using multi-factorial ANOVAs with litter size, sex, adult diet, and LPS treatment as between factors where appropriate, with Tukey *post-hoc* comparisons where significant main effects or interactions were found. We also included time (min) as a repeated measure in analysis of plasma glucose concentrations. Data are presented as the mean + standard error of the mean (SEM). Statistical significance was assumed when *P* ≤ 0.05. Statistical details are reported in the figure legends.

## Results

### Weight gain with neonatal overfeeding

**Figure 1:**
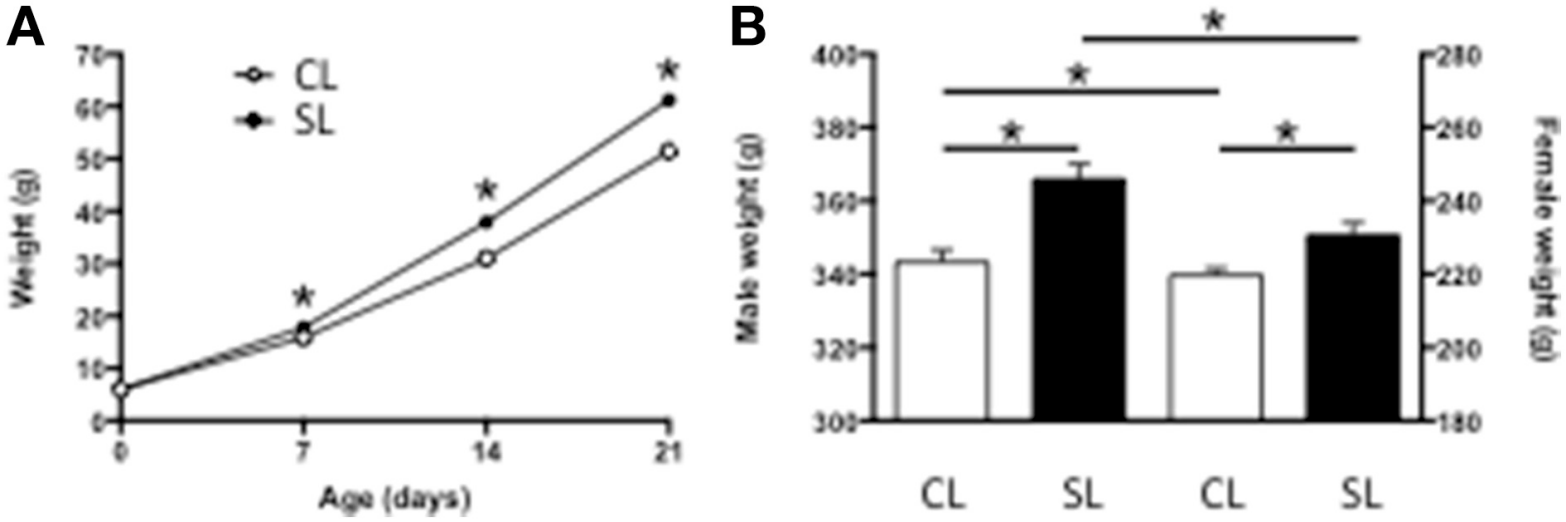
**Effects of neonatal overfeeding on body weight. (A)** Pre-weaning (age, litter size interaction [*F*_(3, 78)_ = 28.83, *P* < 0.001] and **(B)** Adult (P56; significant effect of litter size [*F*_(1, 84)_ = 25.96, *P* < 0.001] and sex [*F*_(1, 84)_ = 1559.46, *P* < 0.001] body weights of rats raised in control (CL) and small (SL) litters. Data are mean + SEM. ^*^P < 0.05.

As we, and others, have previously reported (Spencer and Tilbrook, 2009; Clarke et al., 2012; Ziko et al., 2014), being suckled in SL leads to accelerated weight gain and this is maintained into adulthood compared with rats from CL. Thus, being raised in SL led to pups being significantly heavier by as early as P7 and this was maintained throughout the suckling period (Figure 1A) and into adulthood (Figure 1B).

### Weight gain, food intake, and caloric efficiency with high fat diet in adulthood

**Figure 2:**
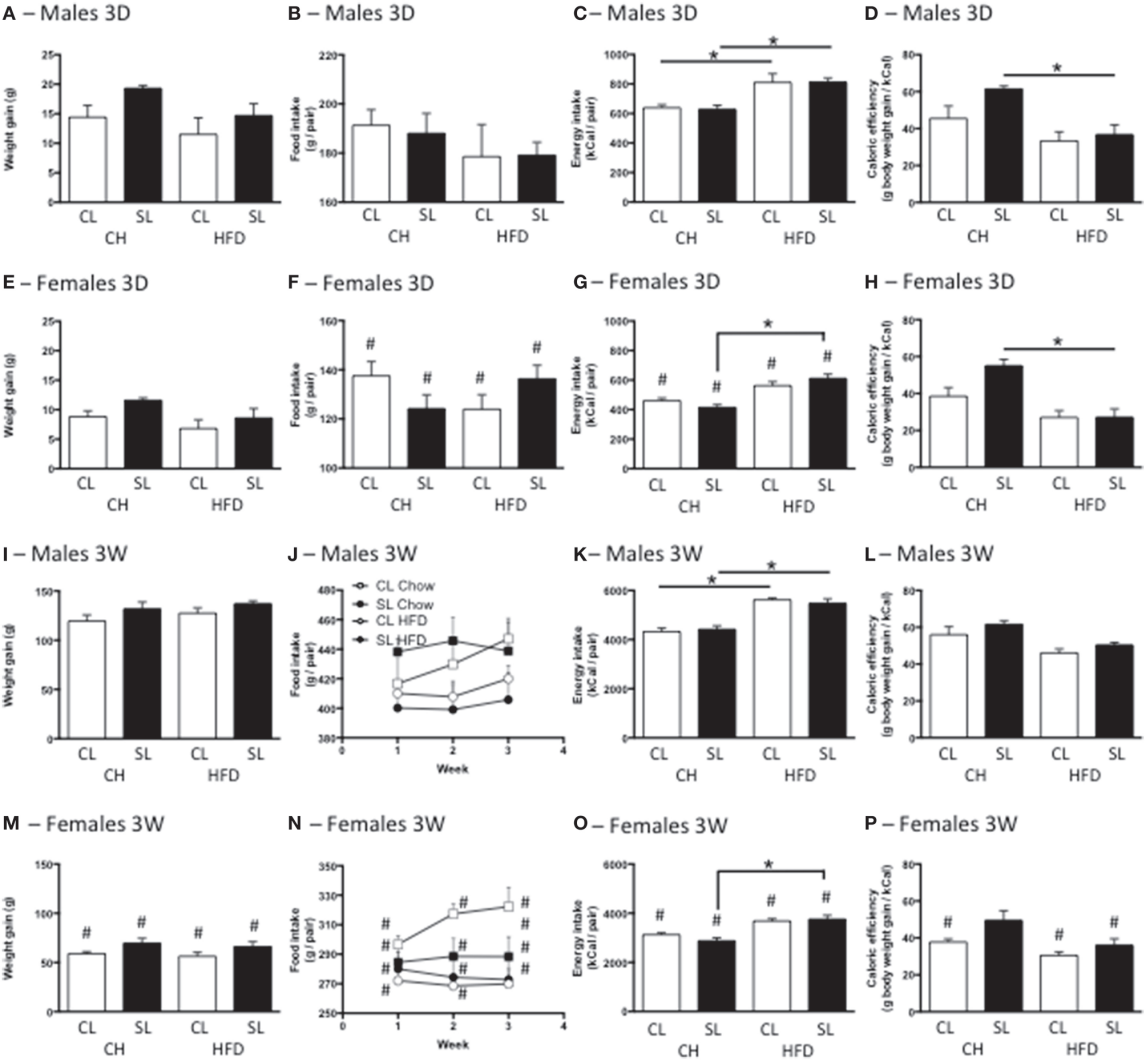
**Effects of neonatal overfeeding on weight gain and food intake after 3 day or 3 week high fat diet. (A,E,I,M)** Weight gain with 3 day (3D; **A,E**) and 3 week (3W; **I,M**) high fat diet (HFD) or chow (CH) in male **(A,I)** and female **(E,M)** adult rats that were raised in control (CL) and small (SL) litters. 3D HFD: significant effect of sex [*F*_(1, 48)_ = 26.77, *P* < 0.001] and diet [*F*_(1, 48)_ = 7.16, *P* = 0.01]. 3W HFD: significant effect of sex [*F*_(1, 50)_ = 374.35, *P* < 0.001] and litter size [*F*_(1, 50)_ = 9.78, *P* = 0.003]. **(B,F,J,N)** Food intake with 3D **(B,F)** and 3W **(J,N)** HFD in male **(B,J)** and female **(F,N)** rats. 3D HFD: significant effect of sex [*F*_(7, 48)_ = 110.74, *P* < 0.001]. 3W HFD: significant effect of sex [*F*_(7, 46)_ = 285.23, *P* < 0.001] and diet [*F*_(7, 46)_ = 12.03, *P* = 0.001]. **(C,G,K,O)** Energy intake with 3D **(C,G)** and 3W **(K,O)** HFG in male **(C,K)** and female **(G,O)** rats. 3D HFD: significant effect of sex [*F*_(7, 45)_ = 88.54, *P* < 0.001], and diet [*F*_(7, 45)_ = 53.58, *P* < 0.001]. 3W HFD: significant effect of sex [*F*_(7, 46)_ = 266.19, *P* < 0.001], and diet [*F*_(7, 46)_ = 93.34 *P* < 0.001), significant sex × diet interaction [*F*_(7, 46)_ = 5.54, *P* = 0.023]. **(D,H,L,P)** Caloric efficiency with 3D **(D,H)** and 3W **(L,P)** HFD in male **(D,L)** and female **(H,P)** rats. 3D HFD: significant effect of litter size [*F*_(7, 45)_ = 6.83, *P* = 0.012], sex [*F*_(7, 45)_ = 4.53, *P* = 0.039], and diet [*F*_(7, 45)_ = 30.73, *P* < 0.001], significant litter size × diet interaction [*F*_(7, 45)_ = 4.45, *P* = 0.041]. 3W HFD: significant effect of litter size [*F*_(7, 46)_ = 10.75, *P* = 0.002], sex [*F*_(7, 46)_ = 51.85, *P* < 0.001], and diet [*F*_(7, 46)_ = 25.39, *P* < 0.001]. Data are mean + SEM. ^#^Sex difference between corresponding groups. ^*^As indicated, *P* < 0.05.

Neonatal overfeeding did not cause significant differences in the weight gained with the 3D high fat diet in males or females (Figures 2A,E). There were significant effects of sex and diet, with females gaining less weight over the period than males, and those on high fat diet gaining less weight than those on standard rat chow, but there were no differences between relevant groups with *post-hoc* comparisons. After 3W of high fat diet, all female groups had gained less weight than all male groups. There was also an effect of litter size, with SL gaining more weight than CL but no differences between relevant groups with *post-hoc* comparisons (Figures 2I,M).

Consistent with their size, females ate less than males in both the 3D and 3W analyses. There was also a significant effect of diet on food intake after 3W, with high fat diet-fed rats eating fewer grams of food than standard chow-fed rats, in total and for each of the 3 weeks (Figures 2B,F,J,N).

Calculations of total energy consumption revealed the high fat diet groups consumed more energy than the chow groups at 3D and 3W, and males ate more than females. However, there was no influence of neonatal overfeeding on total energy consumption (Figures 2C,G,K,O).

Caloric efficiency is a measure of the ability to convert calories into body weight. Thus, a reduced caloric efficiency reflects the need to consume more calories to maintain body weight. 3D high fat diet significantly reduced caloric efficiency in SL but not CL male and female rats (Figures 2D,H). The 3W high fat diet significantly reduced caloric efficiency in SL but not CL females (Figures 2L,P).

### Fat mass and triglyceride content with high fat diet in adulthood

**Figure 3:**
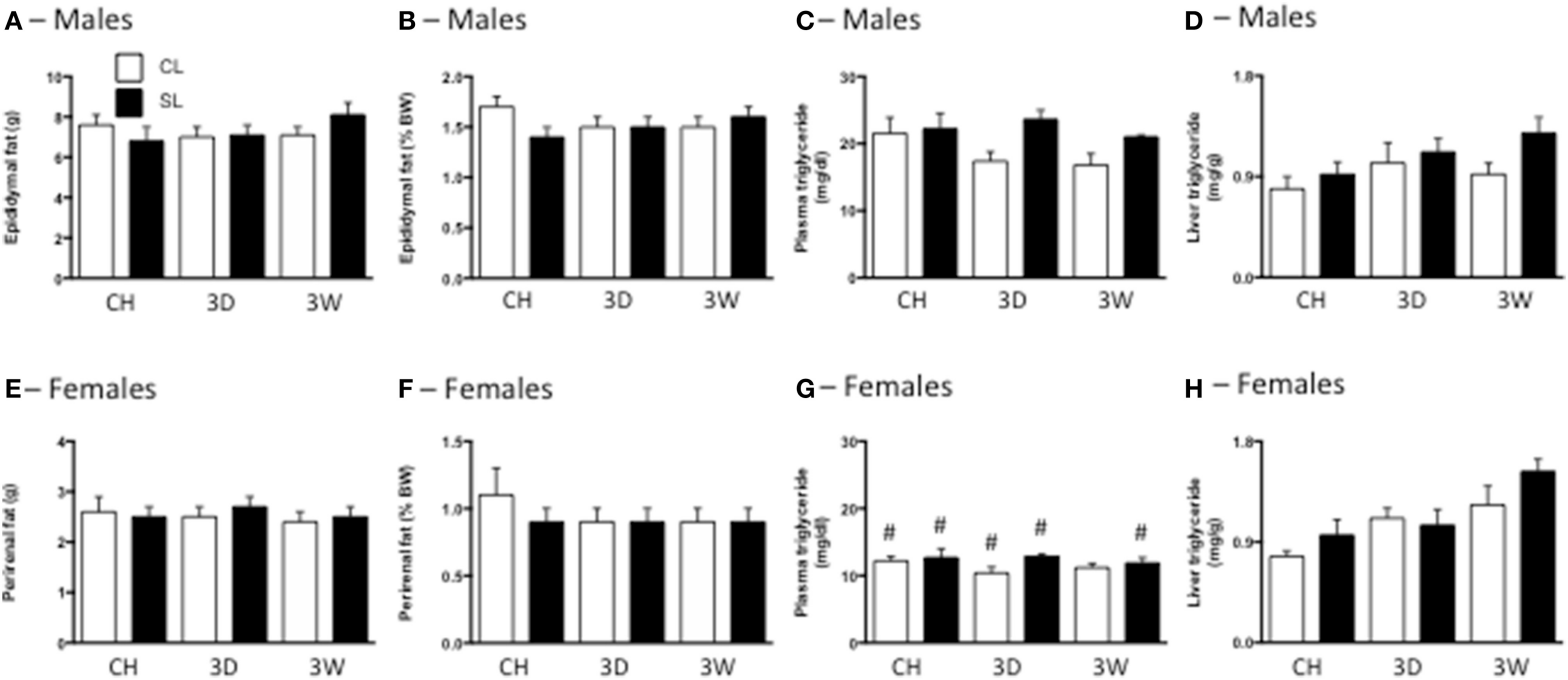
**Effects of neonatal overfeeding on fat mass and triglyceride content after 3 day or 3 week high fat diet**. Total fat **(A,E)** and percentage fat **(B,F)** after 3 day (3D) and 3 week (3W) high fat diet or chow (CH) in male **(A,B)** and female **(E,F)** adult rats that were raised in control (CL) and small (SL) litters. **(C,G)** Plasma triglycerides in male **(C)** and female **(G)** rats. Significant effect of litter size [*F*_(11, 91)_ = 4.64, *P* = 0.034], and sex [*F*_(11, 91)_ = 81.91, *P* < 0.001]. **(D,H)** Liver triglycerides in male **(D)** and female **(H)** rats. Significant effect of litter size [*F*_(11, 72)_ = 4.24, *P* = 0.043] and diet [*F*_(11, 72)_ = 5.69, *P* = 0.001]. Data are mean + SEM. ^#^Sex difference between corresponding groups. *P* < 0.05.

Surprisingly, there were also no differences in total or percentage fat between any of the CL and SL groups (Figures 3A,B,E,F). We did not make a sex comparison in this analysis since the fat pads were different. There was a significant effect of litter size on plasma triglyceride concentrations, with generally increased triglyceride levels in rats from SL. There was also an effect of sex, with females of each group having lower triglyceride levels than their male counterparts (Figures 3C,G). We also detected significant effects of litter size and diet on liver triglyceride concentrations, with SL and the high fat diets increasing these levels (Figures 3D,H).

### Glucose utilization with high fat diet in adulthood

**Figure 4:**
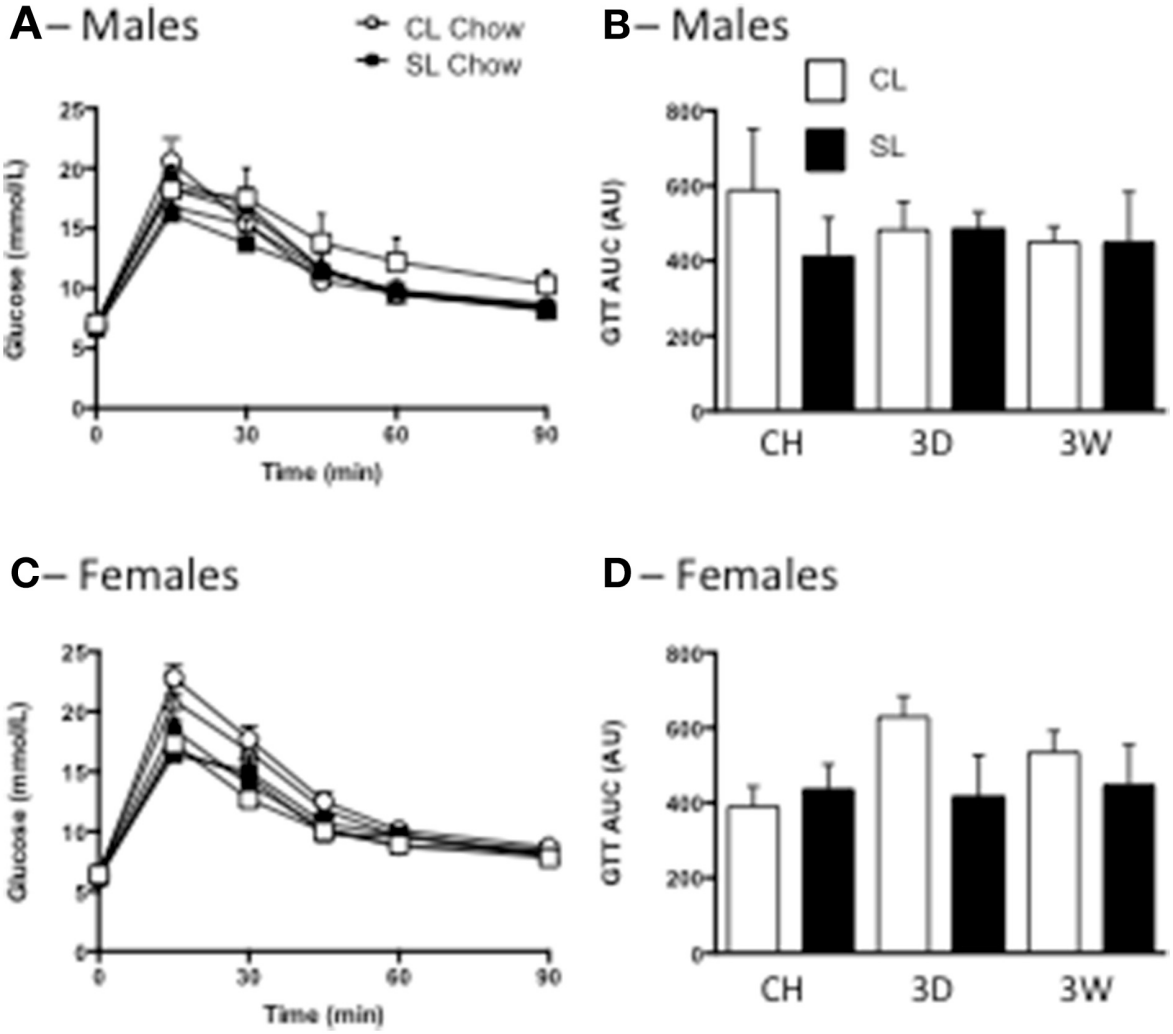
**Effects of neonatal overfeeding on glucose utilization after 3 day or 3 week high fat diet**. Glucose concentrations **(A,C)** and incremental area under the curve (iAUC) responses **(B,D)** to an i.p. glucose tolerance test after 3 day (3D) and 3 week (3W) high fat diet or chow (CH) in male **(A,B)** and female **(C,D)** adult rats that were raised in control (CL) and small (SL) litters. Data are mean + SEM. There were no differences between groups.

In accordance with the minimal effects of the high fat diet seen on overt measures of weight gain and adiposity, we also saw no significant differences in fasting glucose levels, or tolerance to glucose among the groups in males or females (Figure 4).

### Peripheral inflammation with high fat diet in adulthood; gene expression

**Figure 5:**
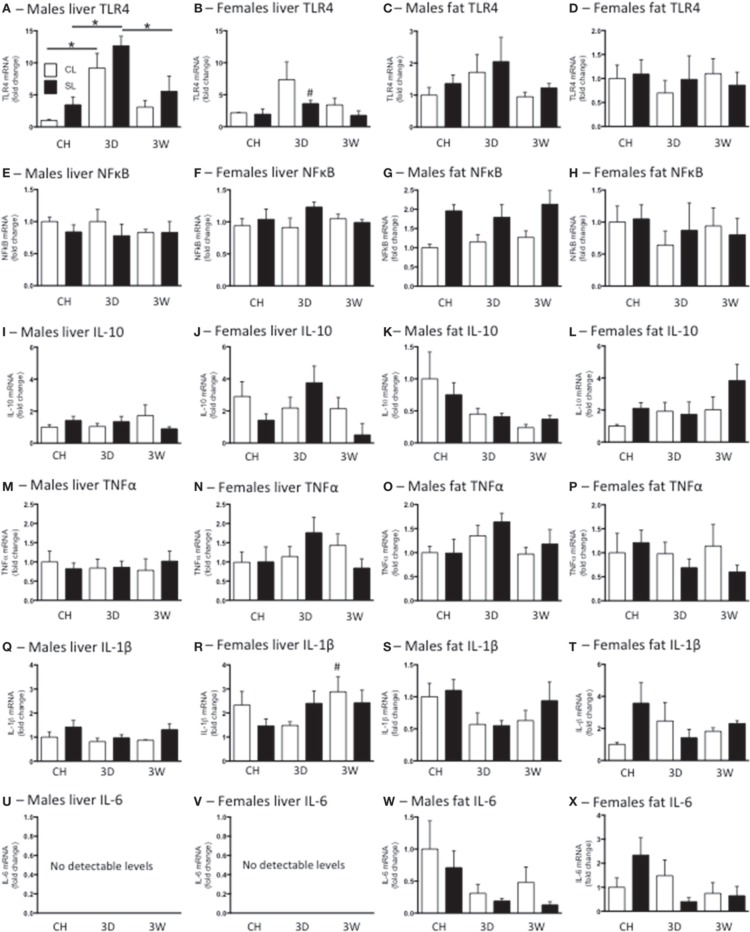
**Effects of neonatal overfeeding on peripheral inflammatory gene expression after 3 day or 3 week high fat diet**. Liver and fat TLR4 **(A–D)**, NFκ B **(E–H)**, interleukin (IL)-10 **(I–L)**, TNFα **(M–P)**, IL-1β **(Q–T)** and IL-6 **(U–X)** after 3 day (3D) and 3 week (3W) high fat diet or chow (CH) in male and female adult rats that were raised in control (CL) and small (SL) litters. Liver TLR4: significant effect of diet [*F*_(11, 60)_ = 18.71, *P* < 0.001] and sex [*F*_(11, 60)_ = 8.25, *P* = 0.006], significant litter size × sex interaction [*F*_(11, 60)_ = 7.47, *P* = 0.008], significant diet x sex interaction [*F*_(11, 60)_ = 3.39, *P* = 0.04]. Liver NFκ B: significant effect of sex [*F*_(11, 56)_ = 4.12, *P* = 0.047]. Male fat NFκ B: significant effect of litter size [*F*_(5, 33)_ = 14.80, *P* = 0.001]. Liver IL-10 significant effect of sex [*F*_(11, 57)_ = 11.25, *P* = 0.001]. Male fat IL-10 significant effect of diet [*F*_(5, 30)_ = 4.81, *P* = 0.015]. Liver IL-1β significant effect of sex [*F*_(11, 59)_ = 26.42, *P* < 0.001]. Male fat IL-1β significant effect of diet [*F*_(5, 30)_ = 3.29, *P* = 0.051]. Data are mean + SEM. ^#^Sex difference between corresponding groups. ^*^As indicated, *P* < 0.05.

We have previously reported neonatal overfeeding influences peripheral and central immune profiles (Clarke et al., 2012; Ziko et al., 2014). We therefore tested if neonatal overfeeding exacerbates the peripheral and central response of inflammatory markers to high fat diet. In the liver there was an increase in TLR4 mRNA after 3D high fat diet in both CL and SL males compared with their chow-fed counterparts. Interestingly, this increase in TLR4 did not persist, but had returned toward baseline values after 3W (Figure 5A). There were no significant differences between the female groups with *post*-*hoc* tests and no sex differences, but CL females did show a tendency to have elevated TLR4 after 3D high fat diet compared with chow-fed females (Figure 5B).

In liver there was a significant effect of sex on NFκ B, IL-10, and IL-1β mRNA, with females expressing more of these three genes than males, but there were no significant differences with *post*-*hoc* tests except in that there was more IL-1β in females after 3W high fat diet than in males. There were no differences between the groups in liver TNFα mRNA and IL-6 was undetectable in this tissue (Figure 5).

We analyzed male epididymal and female perirenal fat separately as the fat was taken from different regions. There was a significant effect of litter size on fat NFκ B in the males, with SL having more NFκ B than CL, but there were no significant differences between the individual groups with *post*-*hoc* tests (Figure 5G). There was also a significant effect of diet on male IL-10 and IL-1β with the high fat diets reducing expression of these cytokines, but again there were no *post*-*hoc* differences and no further significant differences in male or female fat TLR4, NFκ B, TNFα, or IL-6 mRNA (Figure 5).

### Peripheral inflammation with high fat diet and LPS in adulthood; liver protein

Analysis of liver concentrations of a suite of pro- and anti-inflammatory cytokines revealed no notable effects of diet at 3D or 3W in any of the groups, and no notable effects of the litter size except where IL-2 was suppressed in SL relative to CL. LPS significantly increased liver IL-1α, IL-1β, IL-6, and TNFα across the groups, but there were no significant differences with the *post*-*hoc* tests except in IL-1α CL after 3D high fat diet. We also found significant sex differences, with less of all the cytokines measured in females than in males except IL-1α (Table 1).

**Table 1:** **Liver cytokine (pg/mL) responses to lipopolysaccharide (LPS) after 3 days (3D) and 3 weeks (3W) in rats that were raised in control (CL) or small (SL) litters**.

	**CL**	**SL**	**CL**	**SL**	**CL**	**CL**	**CL**	**SL**	**CL**	**SL**	**CL**	**CL**	**Main effects**
	**CH**	**CH**	**3D**	**3D**	**3W**	**3W**	**CH**	**CH**	**3D**	**3D**	**3W**	**3W**	** **
	**Sal**	**Sal**	**Sal**	**Sal**	**Sal**	**Sal**	**LPS**	**LPS**	**LPS**	**LPS**	**LPS**	**LPS**	
**MALES**													
IL-1α	58.1	40.9	50.9	44.1	70.3	41.5	247.5	188.4	206.9	190.7	186.5	116.1	LPS
	(10)	(6)	(5)	(5)	(11)	(6)	(88)	(79)	(60)^*^	(75)	(68)	(31)	
IL-1 β	807.7	543.5	682.6	614.9	896.1	626.0	1533.3	1453.8	1559.5	1487.7	1643.0	1114.6	LPS, SEX
	(120.2)	(66)	(65)	(71)	(132)	(32)	(260)	(358)	(282)	(463)	(455)	(242)	
IL-2	174.2	129.3	155.4	132.9	177.3	142.2	164.2	150.4	148.8	151.3	166.7	116.8	LITTER, SEX
	(23)	(17)	(14)	(10)	(13)	(6)	(6)	(22)	(13)	(34)	(21)	(15)	
IL-4	1018.5	843.0	884.5	682.0	1040.4	853.6	856.6	1031.4	721.0	1163.8	1081.3	738.1	SEX
	(190)	(309)	(297)	(182)	(336)	(195)	(232)	(341)	(185)	(516)	(415)	(212)	
IL-5	1205.4	966.9	1056.8	905.0	1163.2	1034.3	1007.9	1151.6	879.10	1160.0	1099.5	964.1	SEX
	(167)	(185)	(223)	(142)	(253)	(163)	(175)	(266)	(100)	(311)	(247)	(181)	
IL-6	84.9	57.4	72.7	56.2	88.3	68.8	106.0	102.5	94.2	109.1	103.7	71.4	LPS, SEX
	(12)	(12)	(10)	(7)	(10)	(11)	(14)	(29)	(18)	(36)	(26)	(10)	
IL-10	4885.6	3760.2	4331.9	3655.3	5228.0	4326.8	4764.5	4952.8	4140.0	5372.0	5021.6	3824.8	SEX
	(654)	(896)	(794)	(472)	(1058)	(660)	(699)	(1105)	(567)	(1667)^#^	(1108)	(680)	
IL-12	341.2	260.9	277.0	224.4	314.5	257.9	263.5	311.1	233.2	316.1	289.9	249.0	SEX
	(59)	(64)	(84)	(55)	(94)	(61)	(61)	(84)	(49)	(112)	(95)	(61)	
TNF- α	205.7	157.0	173.9	158.8	221.3	168.6	287.2	232.4	243.8	257.9	284.7	194.2	LPS, SEX
	(31)	(36)	(22)	(19)	(20)	(23)	(47)	(58)	(45)	(67)	(77)	(20)	
**FEMALES**													
IL-1 α	40.7	35.6	44.1	37.7	34.5	68.0	155.3	179.0	292.0	136.2	151.8	167.4	
	(3)	(5)	(5)	(4)	(2)	(14)	(41)	(28)	(71)	(35)	(26)	(14)	
IL-1 β	424.1	324.3	459.6	390.7	377.0	524.9	975.4	1328.1	1403.6	930.8	964.0	1084.8	
	(54)	(56)	(45)	(35)	(30)	(76)	(172)	(227)	(241)	(157)	(166)	(113)	
IL-2	122.1	93.5	126.1	111.6	107.2	111.6	102.9	100.4	105.3	93.5	106.3	119.1	
	(7)	(10)	(12)	(11)	(8)	(8)	(14)	(11)	(9)	(13)	(7)	(14)	
IL-4	406.5	313.9	586.7	414.1	369.1	465.9	366.1	287.8	271.4	405.7	350.1	498.3	
	(56)	(88)	(133)	(116)	(59)	(72)	(96)	(49)	(43)	(83)	(48)	(129)	
IL-5	440.0	399.3	526.6	417.1	423.0	563.7	405.4	412.3	354.7	427.3	397.2	500.8	
	(36)	(37)	(50)	(37)	(25)	(69)	(58)	(47)	(35)	(59)	(13)	(61)	
IL-6	56.0	41.7	55.0	46.3	42.5	51.7	62.8	68.9	81.7	55.1	55.7	74.3	
	(7)	(6)	(6)	(5)	(4)	(7)	(8)	(10)	(16)	(9)	(5)	(10)	
IL-10	2206.2	1798.6	2223.9	1995.1	2021.2	2009.6	1849.1	1865.4	2002.2	1891.4	2043.1	2212.5	
	(116)	(162)	(150)	(226)	(165)	(85)	(226)	(143)	(176)	(162)	(84)	(128)	
IL-12	69.8	50.7	80.9	65.5	61.1	88.0	60.7	67.1	52.8	65.8	60.6	82.1	
	(7)	(9)	(11)	(8)	(6)	(14)	(11)	(13)	(7)	(11)	(5)	(13)	
TNF- α	162.2	124.4	129.2	129.0	105.1	133.0	159.4	182.2	203.0	142.0	145.9	182.8	
	(27)	(19)	(15)	(21)	(13)	(25)	(23)	(32)	(52)	(25)	(13)	(32)	

*Interleukin (IL)-1α: significant effect of LPS [F_(23, 118)_ = 70.78, P < 0.001]. IL-1β: significant effect of LPS [F_(23, 117)_ = 77.74, P < 0.001]; significant effect of sex [F_(23, 117)_ = 14.28, P < 0.001]. IL-2: significant effect of litter size [F_(23, 119)_ = 8.47, P = 0.004]; significant effect of sex [F_(23, 119)_ = 52.93, P < 0.001]. IL-4: significant effect of sex [F_(23, 120)_ = 34.36, P < 0.001]. IL-5: significant effect of sex [F_(23, 120)_ = 101.25, P < 0.001]. IL-6: significant effect of LPS [F_(23, 120)_ = 15.14, P < 0.001]; significant effect of sex [F_(23, 120)_ = 22.68, P < 0.001]. IL-10: significant effect of sex [F_(23, 120)_ = 93.59, P < 0.001]. IL-12: significant effect of sex [F_(23, 120)_ = 95.10, P < 0.001]. Tumor necrosis factor (TNF)α: significant effect of LPS [F_(23, 120)_ = 14.08, P < 0.001]; significant effect of sex [F_(23, 120)_ = 20.84, P < 0.001]. Data are mean (SEM)*.

* *Versus saline group*.

# *Versus female group. P < 0.05*.

### Microglial changes with high fat diet in adulthood

**Figure 6:**
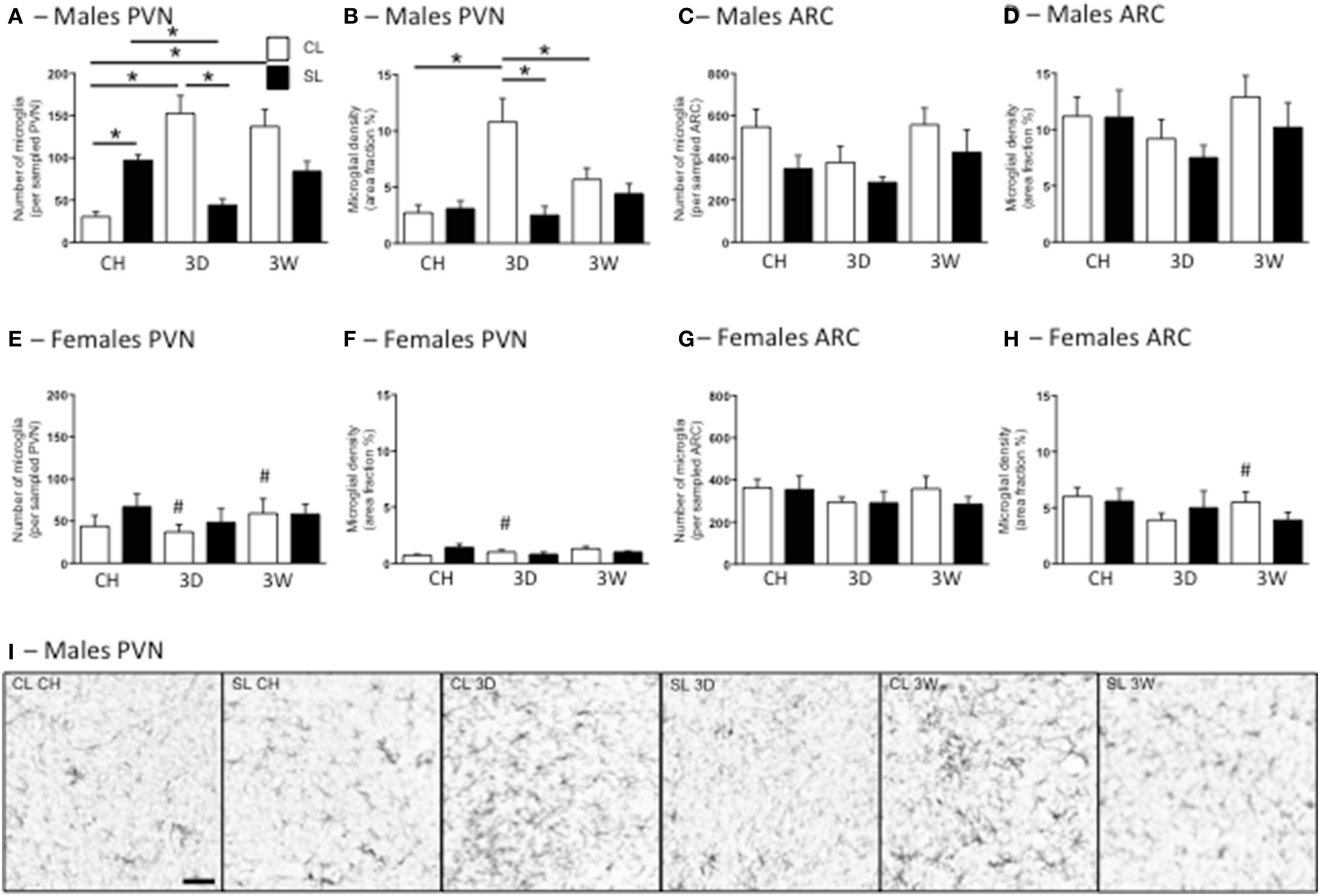
**Effects of neonatal overfeeding on hypothalamic microglia after 3 day or 3 week high fat diet**. Numbers **(A,C,E,G)** and density **(B,D,F,H)** of ionized calcium-binding adapter molecule-1 (Iba-1)-stained cells after 3 day (3D) and 3 week (3W) high fat diet or chow (CH) in male **(A–D)** and female **(E–H)** adult rats that were raised in control (CL) and small (SL) litters. **(A,B,E,F)** paraventricular nucleus of the hypothalamus (PVN). Microglial number: significant effect of sex [*F*_(11, 68)_ = 20.53, *P* < 0.001] and significant litter size × diet × sex interaction [*F*_(11, 68)_ = 7.06, *P* = 0.002]. Microglial density: significant effect of litter size [*F*_(11, 68)_ = 43.68, *P* = 0.029] and sex [*F*_(11, 68)_ = 31.96, *P* < 0.001] and a significant litter size x diet x sex interaction [*F*_(11, 68)_ = 3.54, *P* = 0.035]. **(C,D,G,H)** arcuate nucleus (ARC). Microglial number: significant effect of litter size [*F*_(11, 68)_ = 5.39, *P* = 0.023] and sex [*F*_(11, 68)_ = 7.28, *P* = 0.009]. There was also an effect of diet of *P* < 0.06 [*F*_(11, 68)_ = 2.95, *P* = 0.059]. Microglial density: significant effect of sex [*F*_(11, 68)_ = 38.74, *P* < 0.001]. Data are mean + SEM. ^#^Sex difference between corresponding groups. ^*^As indicated, *P* < 0.05. **(I)** Representative photomicrographs of the PVN from male CL chow, SL chow, CL 3D, SL 3D, CL 3W, and SL 3W illustrating differences in numbers and density of Iba-1-stained cells. Scale bar = 1 mm.

In agreement with our previous findings (Ziko et al., 2014), neonatal overfeeding significantly increased PVN microglial numbers so that under chow-fed conditions, male SL rats had more microglia than CL in this region (Figures 6A,I). In males, the 3D high fat diet caused a substantial increase in microglial numbers and density in CL rats but, interestingly, caused a reduction in microglial numbers in SL rats compared with the chow diet (Figures 6A,I). After 3W high fat diet, microglial numbers remained elevated in CL compared with under chow conditions, but there was no effect of the 3W diet on SL rats (Figures 6A,I). Similar trends were seen in microglial density. In this case, the 3D high fat diet increased microglial density in CL but not SL and the 3W high fat diet had little effect (Figure 6B). In females the responses were more ambiguous, with neonatal overfeeding and adult diet having no significant effects (Figures 6E,F).

In the ARC there were significant effects of litter size, diet, and sex on microglial numbers, with females having fewer microglia and neonatal overfeeding reducing microglial numbers overall, but there were no relevant differences with *post*-*hoc* comparisons (Figures 6C,G). There was also a significant effect of sex on microglial density in this region with *post*-*hoc* tests revealing female CL but not SL rats had reduced microglial density compared with males after 3W high fat diet (Figures 6D,H).

### Neuronal activation with high fat diet in adulthood

**Figure 7:**
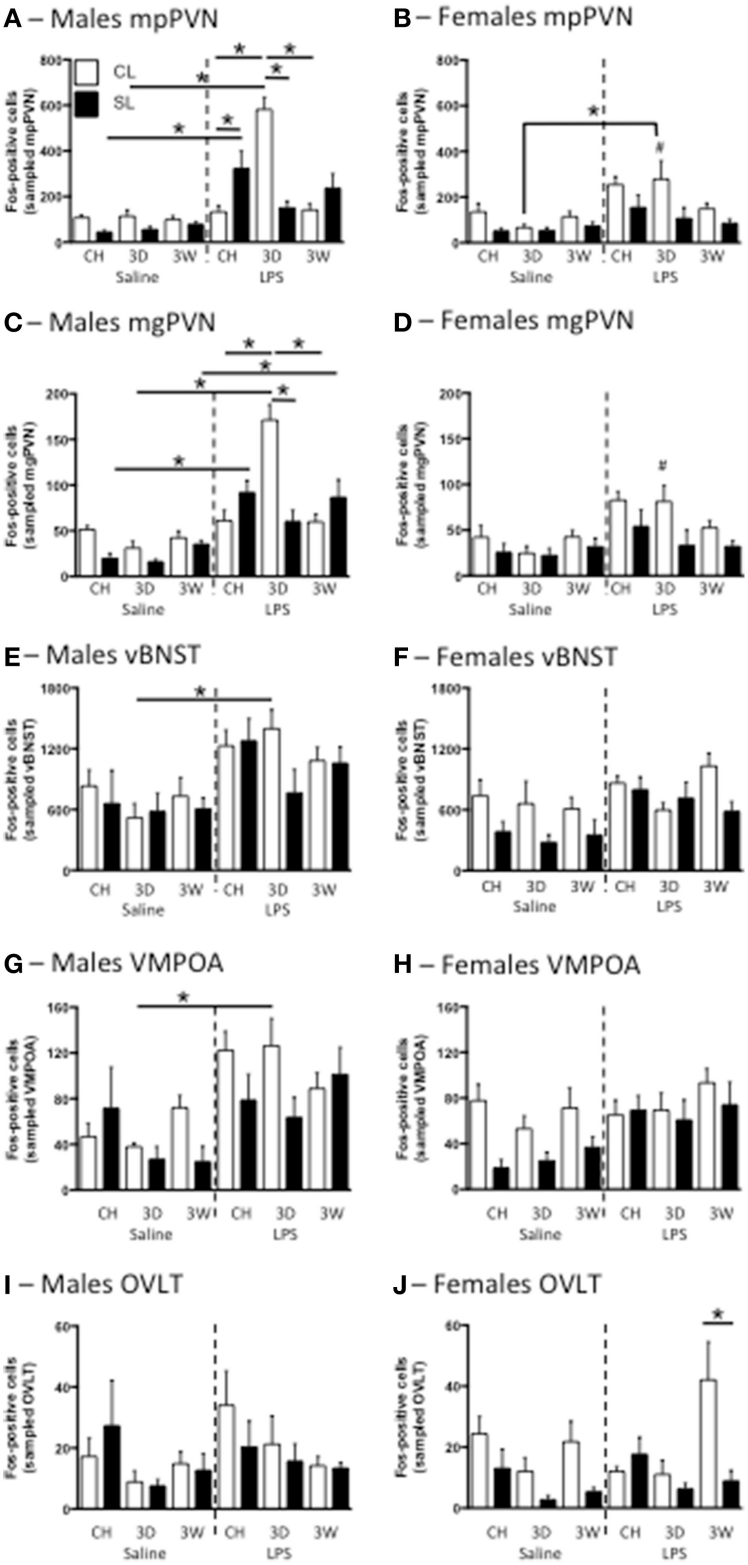
**Effects of neonatal overfeeding on neuronal activation in response to LPS**. Neuronal activation in the medial parvocellular (mp) **(A,B)** and magnocellular (mg) **(C,D)** paraventricular nucleus of the hypothalamus (PVN), the ventral bed nucleus of the stria terminalis (vBNST) **(E,F)**, the ventromedial preoptic area (VMPOA) **(G,H)** and the vascular organ of the laminar terminalis (OVLT) **(I,J)** with LPS after 3 day (3D) and 3 week (3W) high fat diet or chow (CH) in male **(A,C,E,G,I)** and female **(B,D,F,H,J)** adult rats that were raised in control (CL) and small (SL) litters. mpPVN: significant effect of litter size [*F*_(23, 140)_ = 15.09, *P* < 0.001], LPS [*F*_(23, 140)_ = 68.11, *P* < 0.001], diet [*F*_(23, 140)_ = 3.99, *P* = 0.02], and sex [*F*_(23, 140)_ = 7.64, *P* = 0.006] and a significant litter size, LPS, diet, sex interaction [*F*_(23, 140)_ = 5.22, *P* = 0.007]. mgPVN: significant effect of litter size [*F*_(23, 140)_ = 16.85, *P* < 0.001], LPS [*F*_(23, 140)_ = 71.26, *P* < 0.001], and sex [*F*_(23, 140)_ = 12.35, *P* = 0.001] and a significant litter size, LPS, diet, sex interaction [*F*_(23, 140)_ = 3.78, *P* = 0.025]. vBNST: significant effect of sex [*F*_(23, 131)_ = 15.76, *P* < 0.001], LPS [*F*_(23, 131)_ = 31.73, *P* = 0.051], and litter size [*F*_(23, 131)_ = 8.05, *P* = 0.005] and a significant litter size, LPS × diet × sex interaction [*F*_(23, 131)_ = 3.05, *P* = 0.051]. VMPOA: significant effect of LPS [*F*_(23, 130)_ = 31.81, *P* < 0.001] and litter size [*F*_(23, 130)_ = 11.70, *P* = 0.001] as well as a significant litter size x LPS × diet × sex interaction [*F*_(23, 130)_ = 3.91, *P* = 0.023]. OVLT: significant effect of diet [*F*_(23, 133)_ = 5.34, *P* = 0.006] and litter size [*F*_(23, 133)_ = 7.64, *P* = 0.007]. There was also a diet x sex interaction of *P* < 0.06 [*F*_(23, 133)_ = 2.90, *P* = 0.058]. Data are mean + SEM. ^#^Sex difference between corresponding groups. ^*^As indicated, *P* < 0.05.

As previously demonstrated (Clarke et al., 2012), neonatal overfeeding exacerbates the PVN response to LPS in male rats, with SL males having approximately twice as many mp and mg PVN neurons activated after LPS as CL (Figures 7A,C). In male CL rats, the 3D high fat diet led to a markedly increased PVN response to LPS compared with that seen in chow fed CL rats. This response was not seen in the SL group after 3D high fat diet, where there was a tendency for the PVN response to be reduced compared with chow SL. With 3W high fat diet, neuronal activation after LPS was again similar to that seen in chow-fed rats in both CL and SL, but the difference between these two groups was no longer present.

Interestingly, the females had a different profile of Fos expression in response to LPS (Figures 7B,D). Although there were no significant differences between the relevant groups with *post*-*hoc* tests, the trend was for chow-fed SL rats to have a smaller Fos response than CL in both the mp and mg PVN. There was also a trend for the 3W high fat diet to attenuate the response with no effect of the 3D diet. The response to LPS was also significantly higher in males than in females in the CL 3D diet group but, despite an attenuated female response overall compared with males, there were no other significant sex differences with *post*-*hoc* tests. In the dpPVN there were significant main effects of litter size [*F*_(23, 140)_ = 14.86, *P* < 0.001], LPS [*F*_(23, 140)_ = 13.57, *P* < 0.001], and sex [*F*_(23, 140)_ = 49.32, *P* < 0.001] but no relevant differences with *post*-*hoc* comparisons (data not shown).

We also examined neuronal activation in several other brain regions involved in fever regulation and the response to LPS. Although the pattern was not as clear as for the PVN, similar responses were also seen in the vBNST and VMPOA in males, with LPS leading to increased Fos in these regions compared with saline after 3D high fat diet in CL but not SL rats (Figure 7). Specifically, there was an increase in vBNST Fos in LPS-treated 3D CL males compared with saline-treated 3D CL males, but no other relevant differences. In the dBNST there was an LPS, sex interaction [*F*_(23, 126)_ = 4.54, *P* = 0.035], a litter size, sex interaction [*F*_(23, 126)_ = 4.45, *P* = 0.037], and a litter size, diet interaction [*F*_(23, 126)_ = 3.76, *P* = 0.026], but there were no differences with *post*-*hoc* tests (data not shown). In the VMPOA there was again a significant increase in Fos in LPS-treated 3D CL males compared with saline-treated 3D CL males, but no other relevant differences. In the MPOA there were effects of LPS [*F*_(23, 130)_ = 4.48, *P* = 0.036] and litter size [*F*_(23, 130)_ = 7.95, *P* = 0.006], but no differences with *post*-*hoc* tests (data not shown). In the OVLT there were no relevant differences with *post*-*hoc* tests except that in females there were more Fos-positive cells with LPS after 3W high fat diet in CL rats than in SL.

## Discussion

The perinatal nutritional environment is important in long-term metabolic programming and, as such, rats that are overfed as neonates by being suckled in small litters show early accelerated weight gain that is maintained into young-adulthood (Spencer and Tilbrook, 2009; Clarke et al., 2012; Stefanidis and Spencer, 2012; Ziko et al., 2014). This model was established as early as the 1960s (McCance, 1962) and these findings have been consistently replicated by several groups (e.g., Plagemann et al., 1992, 2010; Xiao et al., 2007; Chen et al., 2008). Our data now show that despite this weight gain, neonatally overfed rats are only marginally more susceptible to the metabolic/obesigenic effects of a short or medium-term high fat diet. Neonatally overfed rats had an overall increase in plasma and liver triglyceride content on a high fat diet compared with CL rats. They also had a lower caloric efficiency after the high fat diets compared with their chow counterparts, indicating they gained less weight for the same calorie intake, whereas CL rats did not. However, contrary to our hypothesis that SL rats would be more susceptible to the metabolic effects of a high fat diet, they did not eat more, or gain more visceral fat mass, and they did not display early glucose intolerance that might suggest a pre-diabetic profile. They also had no differences in inflammatory gene expression in the liver or fat or in liver cytokine concentrations. We should note that different fat pads can respond differently to dietary influences and a comprehensive analysis of the different depots may still reveal differences between the groups. However, we did not find differences in any other metabolic or inflammatory parameters to suggest this is a strong possibility.

Interestingly, these neonatally overfed rats did show differences in susceptibility to the central pro-inflammatory effects of short-term (3 days) high fat feeding. Thus, 3 days high fat diet led to significant microgliosis in the PVN in male CL rats, but this was not seen in SL. This increase in microglial numbers in the PVN was still evident at the 3 week mark. Similarly, the PVN response to LPS was markedly enhanced in CL rats by 3 days of high fat diet, but not in SL, although this response was resolved to control levels at 3 weeks. These differences were not seen in the females.

In a recent study, Thaler and colleagues have suggested the early (3 day) inflammatory response to a high fat diet is actually an adaptive one and that it is only with longer-term (e.g., 3 weeks) high fat feeding that a maladaptive pro-inflammatory response ensues (Thaler et al., 2012). Thus, at 3 days on a high fat diet, rats and mice in the Thaler study showed hypothalamic microgliosis and an increase in hypothalamic pro-inflammatory gene expression. This profile disappeared by 7 days but returned after 21 days of high fat diet (Thaler et al., 2012). In light of this work, our findings would suggest that the absence of microgliosis or an exacerbated response to LPS after 3 days of high fat diet in SL is maladaptive; reflective of an inability to effectively respond to the high fat diet. However, if this interpretation is correct, one would expect to see differences between the groups at 3 weeks, which we did not see in this study.

In this investigation, we deliberately selected relatively short periods of high fat feeding. Our hypothesis suggested neonatally overfed rats may be more susceptible to a high fat diet and it was therefore essential to give a metabolic challenge mild enough to avoid a ceiling effect. As with the present study, other groups have seen 3 weeks of high fat diet is not usually sufficient to induce overt body weight and fat mass differences. For example, Maric and colleagues have shown 32% calorie by fat diets for 8 weeks do not cause a difference, compared with chow fed, in fat pad weight in Wistar rats and only cause a significant increase in total weight gain if the diet is butter based (and not if it is coconut oil-based) (Maric et al., 2014). Significant metabolic and inflammatory effects of both a 3 day and 3 week high fat diet have been reported (Thaler et al., 2012), suggesting the dietary challenge in this study would be sufficient to induce inflammation and allow us to detect any differences between the neonatally overfed and control rats. However, it is possible that while an initial adaptive response to the high fat diet was evident at 3 days, 3 weeks was insufficient to reveal susceptibility to the metabolic effects of the challenge. If this is the case, we would expect the neonatally overfed rats to respond differently after a longer period of high fat diet. It is interesting that our 3 day high fat diet actually caused an overall reduction in the normal weight increase. This is likely to be related to the novelty of the new diet, since other experimental factors would also have influenced the chow groups. What this means for the inflammatory outcome is unclear, especially since the elevated energy intake at 3 days in the high fat diet-fed groups implies they were consuming the high fat chow as expected and any reductions in weight gain may therefore be due to non-nutrient factors. It is possible an adaptive anti-inflammatory response to acute high fat diet (Thaler et al., 2012) is aided in rodents by food-novelty-related elevations in glucocorticoids, but this possibility remains to be tested.

One of the more interesting findings to come out of the present study is our evidence of central pro-inflammatory changes in the absence of a significant change in the metabolic or peripheral pro-inflammatory profiles. Apart from an increase in liver TLR4 mRNA in both CL and SL groups at 3 days high fat diet, there were no significant changes in peripheral indicators of obesity or inflammation in the tissues we examined. These data support recently published evidence (Thaler et al., 2012; Maric et al., 2014). Although it has long been recognized obesity is associated with peripheral inflammation, including elevated pro-inflammatory cytokines in circulation (Hotamisligil et al., 1995; Hotamisligil, 2006), more recent evidence, and our own from this study, is suggesting central inflammation and neuronal injury with high fat diet actually precedes peripheral inflammation. The systemic inflammatory response to diet or weight gain is derived from excess macrophage infiltration to the adipose tissue and subsequent excess production of pro-inflammatory cytokines. It is likely this is a relatively chronic process and possible it is driven, to a degree, by central inflammation (Weisberg et al., 2003; Xu et al., 2003). Thaler and colleagues have shown markers of inflammation in the hypothalamus are elevated as early as 24 h after the onset of a high fat diet. Within a week this is reflected in neuronal injury. Indices of peripheral inflammation, however, are not evident until weeks to months of the diet (Thaler et al., 2012). Similarly, Maric and colleagues have shown diet high in saturated fat leads to central inflammation in the absence of peripheral even as late as 8 weeks after onset (Maric et al., 2014). Our current findings tend to support these suggestions that central inflammation, at least in terms of microgliosis and susceptibility to an immune challenge, occurs early after the commencement of a high fat diet, and precedes the development of metabolic dysregulation or an obese profile. In this regard, it will be interesting to examine how high fat diet influences acute pro-inflammatory circulating signals such as leptin in these neonatally overfed populations, since adipokines such as leptin are important in influencing central inflammation (Gao et al., 2014).

Our findings also suggest that a short period of high fat diet feeding may actually leave the individual seriously vulnerable to bacterial infection at this time. A hypersensitive HPA axis after 3 days high fat diet may be an adaptive attempt to curtail inflammation through glucocorticoid production (Thaler et al., 2012). However, our CL rats given 3 days high fat diet responded to LPS with a six-fold increase in neuronal activation in the PVN. Although we did not see differences from the chow-fed groups in LPS/fever-regulatory brain regions (VMPOA, OVLT, BNST) and we did not measure fever and sickness behavior directly, a response of this magnitude in the PVN is likely to reflect a more severe illness with LPS (Tarr et al., 2012). Several studies have shown microglia behave differently depending upon their background or basal state. For instance, early life immune challenge can leave microglia “primed” to more readily respond to a similar challenge later on (Bland et al., 2010; Williamson et al., 2011). We have recently shown neonatal overfeeding has a similar effect, with neonatally overfed rats having an exaggerated microglial, febrile, cytokine, and HPA axis response to LPS (Clarke et al., 2012; Ziko et al., 2014). The present work suggests that instead of exacerbating this response, the 3 day and 3 week high fat diets dampen it, at least in terms of PVN neuronal activation after LPS, uncovering the possibility of an interaction between the “primed” microglial state and subsequent diet.

Another notable finding of the present study is that the sexes responded quite differently to the high fat diet. While CL males were affected by 3 days high fat diet in a number of parameters, females were not. We deliberately did not control for cycle stage in our females as this imposes an additional stressor on the animals. However, we believe cycle stage is unlikely to account for these sex differences since the variability in the data was similar for females as for males. Although few investigators have examined both males and females in the same study, our findings do concur with reported literature. For instance, male mice develop insulin resistance after a short period of diet high in saturated or unsaturated fat. Female mice retain their insulin sensitivity with the same diet (Senthil Kumar et al., 2014). Likewise, female rats are relatively protected against the metabolic effects of a high fructose or sucrose diet, whereas males develop insulin resistance and hypertension under the same conditions (Galipeau et al., 2002). Our data thus illustrate female rats are likely to be more resilient to the effects of short-term high fat diet than males. These data also highlight the importance of including both sexes as study subjects, or at least exercising care when extrapolating data from one sex to another.

In summary, rats made overweight by early life overfeeding are unlikely to be substantially more vulnerable to a short-term adult-onset high fat diet than control rats in terms of developing further obesity or a diabetogenic profile. On the other hand, neonatally overfed rats were less responsive to the central pro-inflammatory effects of a 3 day high fat diet than controls. Whether this represents a maladaptive inability to combat the central effects of the high fat diet or, rather, a resilience to the challenge, remains to be determined in future work.

## Author contributions

Guohui Cai, Juan Molero and Sarah J. Spencer conceived of and designed this study. Guohui Cai, Ilvana Ziko, Stanley M. H. Chan, Xiao-Yi Zeng, Songpei Li, Juan Molero, and Sarah J. Spencer ran the animal studies and collected samples. Guohui Cai, Tara Dinan, Joanne M. Barwood, Simone N. De Luca, Alita Soch, and Sarah J. Spencer analyzed samples and interpreted the data. Sarah J. Spencer wrote the manuscript. All authors revised the manuscript critically. All authors give final approval of the version to be published and agree to be accountable for all aspects of the work in ensuring that questions related to the accuracy or integrity of any part of the work are appropriately investigated and resolved.

### Conflict of interest statement

The authors declare that the research was conducted in the absence of any commercial or financial relationships that could be construed as a potential conflict of interest.
